# Karyotypic characterization of *Centromochlus schultzi* Rössel 1962 (Auchenipteridae, Centromochlinae) from the Xingu River basin: New inferences on chromosomal evolution in *Centromochlus*

**DOI:** 10.1590/1678-4685-GMB-2023-0105

**Published:** 2024-03-25

**Authors:** Samantha Kowalski, Chrystian Aparecido Grillo Haerter, Diana Paula Perin, Fábio Hiroshi Takagui, Patrik Ferreira Viana, Eliana Feldberg, Daniel Rodrigues Blanco, Josiane Baccarin Traldi, Lucia Giuliano-Caetano, Roberto Laridondo Lui

**Affiliations:** 1 Universidade Estadual de Londrina, Centro de Ciências Biológicas, Londrina, PR, Brazil.; 2 Universidade Estadual do Oeste do Paraná, Centro de Ciências Biológicas e da Saúde, Cascavel, PR, Brazil.; 3 Instituto Nacional de Pesquisas da Amazônia, Coordenação de Biodiversidade, Manaus, AM, Brazil.; 4 Universidade Tecnológica Federal do Paraná, Santa Helena, PR, Brazil.; 5 Universidade Federal do Amazonas, Departamento de Genética, Manaus, AM, Brazil.

**Keywords:** rDNA, Synteny, ITS, Tatia

## Abstract

Centromochlinae is a widely diverse subfamily with more than 50 species and several taxonomic conflicts due to morphological similarity between *Tatia* and *Centromochlus* species. However, cytogenetic studies on this group have been limited to only four species so far. Therefore, here we present the karyotype of *Centromochlus schultzi* from the Xingu River in Brazil using classic cytogenetic techniques, physical mapping of the 5S and 18S rDNAs, and telomeric sequences (TTAGGG)_n_. The species had 58 chromosomes, simple NORs and 18S rDNA sites. Heterochromatic regions were detected on the terminal position of most chromosomes, including pericentromeric and centromeric blocks that correspond to interstitial telomeric sites. The 5S rDNA had multiple sites, including a synteny with the 18S rDNA in the pair 24st, which is an ancestral feature for Doradidae, sister group of Auchenipteridae, but appears to be a homoplastic trait in this species. So far, *C. schultzi* is only the second species within *Centromochlus* to be karyotyped, but it has already presented characteristics with great potential to assist in future discussions on taxonomic issues in the subfamily Centromochlinae, including the first synteny between rDNAs in Auchenipteridae and also the presence of heterochromatic ITSs that could represent remnants of ancient chromosomal fusions.

## Introduction

The driftwood catfish family, Auchenipteridae, is a monophyletic clade supported by morphological and molecular synapomorphies (Birindelli, 2014; Calegari et al., 2019). This family is composed by 25 genera and 128 valid species (Fricke *et al*., 2023) and is currently divided into two subfamilies: Auchenipterinae, comprising 18 genera and 78 species, and Centromochlinae, with 7 genera and 50 species (Fricke *et al*., 2023). Centromochlinae is the most unstable subfamily from the taxonomic point of view, with the diagnostic limits of some genera still fragilely defined, even after several and recent taxonomic revisions (Calegari *et al*., 2019; Sarmento-Soares and Martins-Pinheiro, 2020).

According to Fricke *et al.* (2023), the genus *Centromochlus* Kner 1858 consists of nine species: *Centromochlus heckelii* (De Filippi 1853), *Centromochlus schultzi* Rössel, 1962, *Centromochlus existimatus* Mees 1974, *Centromochlus musaicus* (Royero 1992), *Centromochlus macracanthus* Soares-Porto 2000, *Centromochlus carolae* (Vari and Ferraris 2013), *Centromochlus melanoleucus* (Vari and Calegari 2014), *Centromochlus orca* Sarmento-Soares, Lazzarotto, Py-Daniel and Leitão 2017, and *Centromochlus akwe* Coelho, Chamon and Sarmento-Soares 2021. However, the *Centromochlus* species are morphologically similar to other genera of Centromochlinae, which historically resulted in several reallocations, mainly involving *Tatia* Miranda-Ribeiro 1911. As a result, establishing taxonomic limits for these species remains a major challenge. For instance, Grant (2015) proposed that *Centromochlus* would consist of four subgenera: *Balroglanis*, *Duringlanis*, *Sauronglanis* and *Ferrarissoaresia*. Calegari *et al*. (2019) elevated *Balroglanis*, *Duringlanis* and *Ferrarissoaresia* to the level of genera and synonymized *Sauronglanis* with *Tatia*. Recently, *Balroglanis* which included *B. schultzi*, *B. macracanthus* and *B. carolae* was synonymized with *Centromochlus* (Sarmento-Soares and Martins-Pinheiro, 2020), and only *Duringlanis* and *Ferrarissoaresia* remains as valid genera (Fricke *et al*., 2023).

The difficulty in determining external morphological characters for delimiting the taxonomic status of *Centromochlus* species interferes with the estimate of diversity of the group and the understanding of its phylogenetic relationships. In similar contexts, cytogenetics has proved to be an important tool, contributing to solve taxonomic and phylogenetic problematics (e.g., Bertollo et al., 2000; Artoni et al., 2015; Santos et al., 2021; Takagui et al., 2021). However, cytogenetic studies in Auchenipteridae are restricted to 12 species, which are distributed in five genera of Auchenipterinae (*Ageneiosus* Lacepède 1803, *Auchenipterus* Bleeker 1862, *Entomocorus* Eigenmann 1917, *Trachelyopterus* Cuvier and Valenciennes 1840, and *Tympanopleura* Eigenmann 1912), and three genera of Centromochlinae (*Centromochlus*, *Tatia* and *Glanidium* Lütken 1874) (Table 1).

**Table 1 -:** Cytogenetic data in Auchenipteridae. 2*n*: diploid number; m: metacentric; sm: submetacentric; st: subtelocentric; a: acrocentric; p: short arm; q: long arm; AM: Amazonas state; GO: Goiás state; PR: Paraná state; MT: Mato Grosso state; MS: Mato Grosso do Sul state; MG: Minas Gerais state; RN: Rio Grande do Norte state; Pará state; NI: ITS not investigated; ND: ITS not detected.

Species	Location	2n	NORs/ 18S rDNA	5S rDNA	ITS	Ref.
**xAuchenipterinae**						
*Ageneiosus inermis* (*cited as *Ageneiosus brevifilis*)	Solimões River basin, Manaus (AM)	56	p, sm	-	NI	Fenocchio and Bertollo (1992)*
	Araguaia River basin, Aragarças (GO)	56	pair 20, p, sm	pair 4, p, m	pair 1, p, m	Lui *et al.* (2013a)
*Auchenipterus nuchalis*	Araguaia River basin, Aragarças (GO)	58	pair 14, p, sm	pair 22, p, st	NI	Machado *et al.* (2021)
*Auchenipterus osteomystax* (cited as *Auchenipterus nuchalis*)	Paraná River basin, Porto Rico (PR)	58	pair 15, p, sm	-	NI	Ravedutti and Júlio Jr (2001)
*Entomocorus radiosus*	Paraguay River basin, Poconé (MT)	58	pair 21, p, st	pair 12, p, sm pair 13, p, sm pair 14, p, sm pair 15, p, sm pair 16, p, sm pair 18, p, st pair 19, p, st	NI	Machado *et al.* (2021)
*Trachelyopterus coriaceus*	Araguaia River basin, São Miguel do Araguaia (GO)	58	pair 23, p, st	pair 3, p, m pair 16, q, sm	NI	Santos et al. (2021); Haerter et al. (2022, 2023)
*Trachelyopterus* aff. *coriaceus* (*cited as *Trachelyopterus* sp.)	Bento Gomes River basin (MT)	58	pair 22, p, st	pair 16, q, sm pair 18, p, sm	ND	Lui *et al.* (2021)*; Haerter et al. (2022, 2023)
*Trachelyopterus galeatus* (*cited as *Parauchenipterus galeatus*)	Paraná River basin, Porto Rico (PR)	58	pair 15, p, sm	-	NI	Ravedutti and Júlio Jr (2001)*
	Paraná River basin, Três Lagoas (MS)	58	pair 25, p, st	pair 16, p, sm pair 17, q, sm	NI	Lui et al. (2010)*
	Piumhi River basin, Capitólio (MG)	58	pair 24, p, st	pair 15, p, sm pair 16, q, sm	NI	Lui et al. (2010)*
	São Francisco River basin, Lagoa da Prata (MG)	58	pair 23, p, st	pair 16, p, sm pair 17, q, sm	ND	Lui et al. (2010)*
	Pium River basin, NE Oriental (RN)	58	p, sm	-	NI	Araújo and Molina (2013)*
	Amazon River basin, Manaus (AM)	58	pair 20, p, st	pair 14, p, sm pair 16, q, sm	ND	Haerter et al. (2022, 2023)
*Trachelyopterus* aff. *galeatus* (*cited as *Parauchenipterus galeatus*)	Araguaia River basin, São Miguel do Araguaia (GO)	58	pair 24, p, st	pair 3, q, m	NI	Santos et al. (2021)*; Haerter et al. (2022, 2023)
*Trachelyopterus porosus*	Amazon River basin, Manaus (AM)	58	pair 23, p, st	pair 3, p, m pair 4, p, m	ND	Haerter et al. (2022, 2023)
*Trachelyopterus striatulus* (*cited as *Parauchenipterus striatulus*)	Doce River basin, Mariléia (MG)	58	pair 23, p, st	pair 10, p, sm pair 13, p, sm pair 15, q, sm	NI	Santos et al. (2021)*; Haerter et al. (2022, 2023)
*Tympanopleura atronasus* (cited as *Ageneiosus atronases*)	Solimões River basin, Manaus (AM)	56	q, sm	-	NI	Fenocchio and Bertollo (1992)
**Centromochlinae**						
*Centromochlus heckelii*	Solimões River, Manaus (AM)	46	pair 20, p, a pair 12, p(W)	-	NI	Kowalski et al. (2020)
*Centromochlus schultzi*	Xingu River basin, Altamira (PA)	58	pair 24, p, st	pair 4, p, m pair 24, p, st pair 27, p, a pair 28, p, a	pair 1, p, m pair 3, c, m	Present study
*Glanidium ribeiroi*	Segredo reservoir, Iguazu River basin (PR)	58	pair 13, p, sm	-	NI	Fenocchio et al. (2008)
	Salto Osório reservoir, Iguazu River basin (PR)	58	pair 13, p, sm	-	NI	Fenocchio et al. (2008)
	Salto Caxias reservoir, Iguazu River basin (PR)	58	pair 17, p, sm	-	NI	Ravedutti and Júlio Jr (2001)
	Iguazu River basin, Capanema (PR)	58	pair 14, p, sm	pair 16, q, sm	ND	Lui *et al.* (2015)
*Tatia jaracatia*	Iguazu River basin, Capanema (PR)	58	pair 28, p, st	pair 4, p, m pair 18, p, sm pair 19, q, sm pair 29, p, sm	NI	Lui *et al.* (2013b)
*Tatia neivai*	Machado River basin, Denise (MT)	58	pair 28, p, st	pair 4, p, sm pair 21, p, sm pair 22, q, sm	NI	Lui *et al.* (2013b)

Considering this context, this work presents the chromosomal analyses of *Centromochlus schultzi* from the Xingu River basin. We aimed to discuss evolutionary aspects of the *C. schultzi* karyotype as well as provide cytotaxonomic markers that may contribute to the discussions about the organization of Centromochlinae.

## Material and Methods

Eight specimens (five females and three males) of *Centromochlus schultzi* were collected in the Xingu River, Altamira region (PA), Brazil (2º53’49’’S; 51º56’09’’W) (Permanent License SISBIO 49379). The specimens were transported to the Instituto Nacional de Pesquisas da Amazônia (INPA), and deposited in the INPA Fish Zoological Collection (INPA/MCTI) (INPA-ICT 059877). The mitotic chromosome suspensions were obtained according to Moreira-Filho and Bertollo (1990) authorized by the Committee on Ethics in Animal Experimentation and Practical Classes of Unioeste (Protocol 09/13 - CEEAAP/Unioeste).

The chromosomes were stained with Giemsa 5% to classify the morphology according to Levan et al. (1964). The constitutive heterochromatin analysis (C-banding) was performed following the protocol described by Sumner (1972), with modifications by Lui et al. (2012). The detection of the Nucleolus Organizing Regions (AgNORs) was realized according to Howell and Black (1980).

Fluorescent *in situ* hybridization (FISH) was performed according to Pinkel et al. (1986) and modifications suggested by Margarido and Moreira-Filho (2008), with 77% of stringency (200ng of each probe, 50% formamide, 10% sulfate dextran, 2xSSC, pH 7.0 - 7.2, 37 ºC overnight). The (TTAGGG)_n_ probe was amplified by PCR (Ijdo et al., 1991) and labeled with tetramethyl-rodhamine-5-dUTP (Roche). The 18S rDNA probes were obtained through Mini-prep of *Prochilodus argenteus* Spix and Agassiz, 1829 (Hatanaka and Galetti Jr, 2004), labeled by Bio-Nick Translation Mix (Roche), detected by antibiotin-avidin-FITC and amplified with biotinylated anti-avidin (Roche). The 5S rDNA probes were obtained through Mini-prep of *Megaleporinus elongatus* Valenciennes, 1850 (Martins and Galetti Jr, 1999), labeled by Dig-Nick Translation Mix (Roche) and detected by antidigoxigenin-rhodamine (Roche). For the double-FISH with telomeric and 5S rDNA probes, the ribosomal 5S DNA was also labeled by Bio-Nick Translation Mix (Roche), detected with antibiotin-avidin-FITC and amplified with biotinylated anti-avidin.

## Results

**Figure 1 -:**
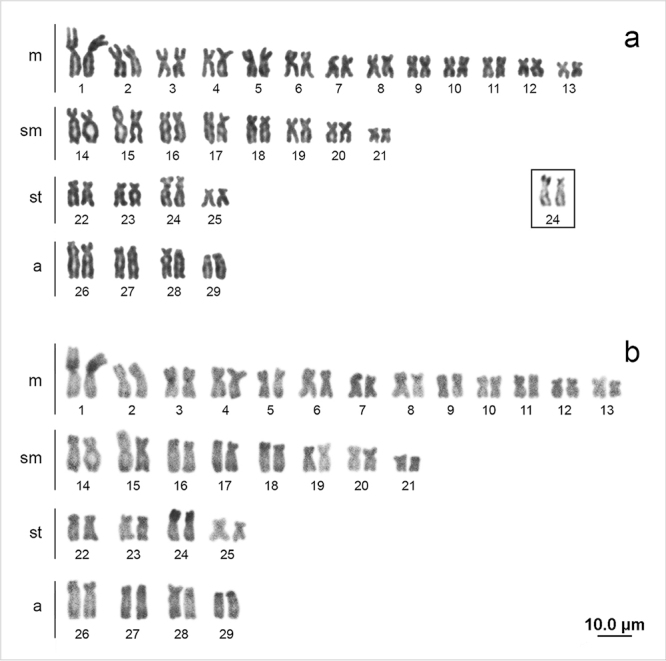
*Centromochlus* *schultzi* karyotype stained with Giemsa **(a)** and submitted to C-banding stained with propidium iodide **(b)**. Ag-NORs are presented in box. There were no chromosomal differences between the sexes.

**Figure 2 -:**
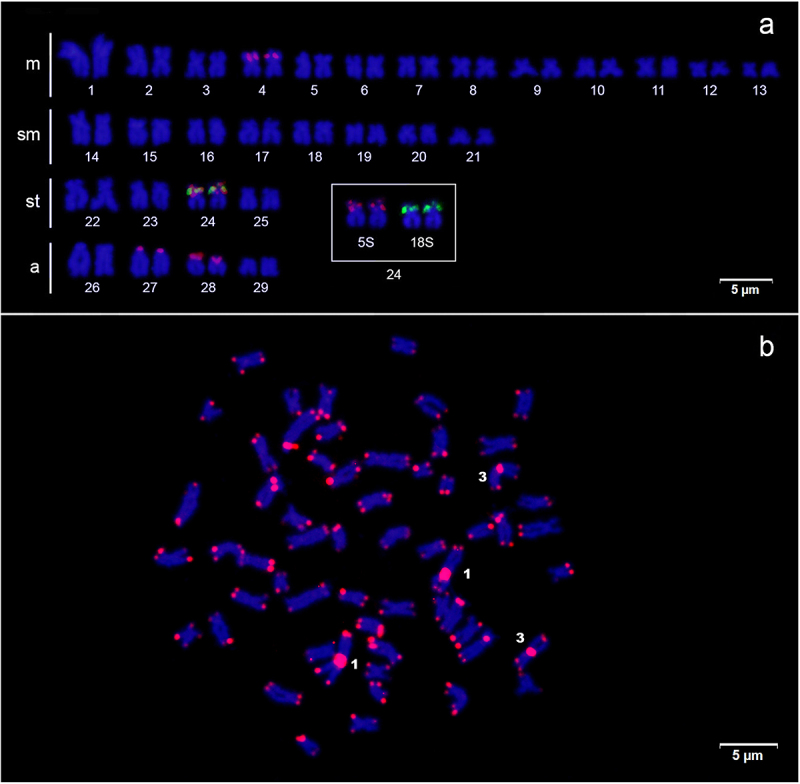
(a) *Centromochlus schultzi* karyotype hybridized with 18S rDNA (green signal on pair 24) and 5S rDNA (red signal on pairs 4, 24, 27 e 28) probes, counterstained with DAPI. (b) *Centromochlus schultzi* metaphase hybridized with telomeric sequence (TTAGGG)_n_. The ITSs are indicated on pairs 1 and 3. There were no chromosomal differences between the sexes.

All chromosomal data described below were the same for both sexes. The diploid number of *Centromochlus schultzi* was 58 chromosomes, organized as 26 metacentric (m), 16 submetacentric (sm), 8 subtelocentric (st) and 8 acrocentric (a), with a fundamental number (FN) of 108 (Figure 1a). Pale sites of heterochromatin were observed in the terminal regions of most chromosomes. It was also observed a large pericentromeric block on the short arm of pair 1m, on the centromere of pair 3m and on the short arm of pair 24st, which also presented the secondary constriction (Figure 1a ), and in the short arm of the chromosomes 18sm and 29a (Figure 1b ). The AgNOR was observed on the interstitial region of the short arm of pair 24 (Figure 1a , box), confirmed by mapping of 18S rDNA (Figure 2a ). The 5S rDNA sites were found on the interstitial region of the short arm of pair 4m, terminal region of the short arm of the pairs 27a and 28a, and also in synteny with the 18S rDNA in the short arm of the pair 24sm (Figure 2a, box). FISH with the telomeric probes (TTAGGG)_n_ evidenced sites in the terminal position of all chromosomes, in addition to non-telomeric sites (ITS - Interstitial Telomeric Site) on the short arm of the pair 1m and on the centromere of the pair 3m (Figure 2b ), coinciding with the location of heterochromatic blocks (Figure 1b ). Double FISH with telomeric and 5S rDNA probes confirmed the lack of synteny between the ITS and the ribosomal DNA (Figure S1).

## Discussion

The few chromosomal data available for Auchenipteridae species show a diploid number of 58 chromosomes in most species (Table 1). Divergent data have been observed in *Ageneiosus* and *Tympanopleura* with 56 chromosomes of the Ageneiosini tribe (Fenocchio and Bertollo, 1992; Lui *et al*., 2013a). This deviation has been attributed to a chromosomal fusion event, as evidenced by the presence of ITS in *Ageneiosus inermis* Linnaeus 1766 (Lui *et al*., 2013a). Another exception is found in *C. heckelii,* which exhibits a diploid number of 46 chromosomes, the lowest diploid number for Auchenipteridae family (Kowalski et al., 2020). These reductions in the number of chromosomes between members of Ageneiosini and *C. heckelii* seem to have originated from independent fusion events, as evidenced by the large phylogenetic distance between them (Kowalski *et al*., 2020). Meyne et al. (1990) presented the first cytogenetic evidence of the presence of ITSs in the karyotypes of different vertebrate species by identifying large blocks of telomeric sequences, preferably located on pericentromeric regions, which have more recently been referred to as heterochromatic ITSs (het-ITSs) (Ruiz-Herrera et al., 2008; Bolzán, 2017). 

ITSs have been described in several fish groups (Ocalewicz, 2013; Vicari et al., 2022); for the Auchenipteridae family, they have been reported only in *A. inermis* (Lui *et al*., 2013a), although there are data of hybridization with telomeric probes in some species of *Trachelyopterus* and a sample of *Glanidium ribeiroi* (Table 1). The occurrence of het-ITSs in chromosomes can be explained through a four-step mechanism: [1] fusion without loss of telomeric sequence; [2] amplification and/or degeneration of these sequences; [3] new chromosome rearrangements; [4] breakage or fission on the heterochromatic site (Ruiz-Herrera et al., 2008). The ITS detected in *C. schultzi* indicate a slightly more complex scenario than that observed in *A. inermis*, which likely only reached the second step, amplification and/or degeneration of these sequences. This is suggested by the fact that *C. schultzi* maintains the common 2n for the family and the position of the ITS in the chromosomes.

The large centromeric ITS blocks (pairs 1m and 3m) observed in *C. schultzi* can potentially be explained through two hypotheses: [1] pericentric inversions followed by telomeric sequence amplification (see Rovatsos et al., 2011); and [2] occurrence of fusions and fissions in different chromosomes during the karyotypic evolution followed by amplification events. Both hypotheses may account for the presence of the ITSs as well as the maintenance of the diploid number. Inversion followed by amplification is an old known event in vertebrate species (see Rovatsos *et al*., 2011), as can be seen in snakes (Viana et al., 2016) and rodent species (Rovatsos *et al*., 2011). In the same way, the presence of these sequences as components of centromeric satellite DNA is also reported in several vertebrate groups (Metcalfe et al., 2004; Nanda et al., 2008; Swier et al., 2012; Bruschi et al., 2014; Viana *et al*., 2016), which may have gone through later amplification events and originated the ITSs in *C. schultzi*. We believe that the mechanism of origin by inversion is more probable, as it is parsimonious in allowing the conservation of the diploid number. If this hypothesis represents a real scenario, this would be the first report in Auchenipteridae.

On the other hand, the cytogenetic study in *C. heckelii* demonstrated 2n=46 chromosomes, showing a large reduction of the diploid number (Kowalski et al., 2020). Alternatively, and less probable, it may indicate that *C. schultzi* would have undergone chromosomal fissions and fusions along its evolutionary history, leading to the formation of ITS that would be sequentially amplified, maintaining the diploid number. This hypothesis considers the proposal of 2n=58 as a plesiomorphic state in Auchenipteridae, or at least in part of the family lineages, as has been deeply investigated and discussed in Doradidae (see Takagui et al., 2021).

In Siluriformes, the presence of ITSs as well as diploid number variation is not a rare event. Fusions have been described in species of some genera, such as *Ageneiosus* (Lui *et al*., 2013a), *Bunocephalus* (Ferreira et al., 2016), *Trachydoras* (Baumgärtner et al., 2016), *Harttia* (Blanco et al., 2013, 2017; Deon et al., 2020) and *Hemiodontichthys* (Carvalho et al., 2018). Centric fissions were described in *Rineloricaria* (Rosa et al., 2012), *Hypostomus* (Traldi et al., 2013) and some *Harttia* species (Deon *et al*., 2020), leading to a probable increase of the diploid number. In Auchenipteridae, the mechanisms of these genetic reorganizations, specifically those we have found in *C. schultzi* still require further analysis.

The common distribution pattern of heterochromatin in Auchenipteridae is terminal pale blocks in most chromosomes (*e.g.*, Lui *et al*., 2013a,b; Machado *et al*., 2021; Santos et al., 2021). *Centromochlus schultzi* exhibited few chromosomal pairs with heterochromatic blocks and the coincidence with the NORs (Figure 1b ) and ITSs (pairs 1m and 3m) sites are worthy of note. In Centromochlinae, stronger heterochromatic markings can be observed on the W chromosome of *C. heckelii* (Kowalski et al., 2020) and in the submetacentric pair 15 of *T. neivai* (Lui *et al*., 2013b). In Auchenipterinae species, pericentromeric markings were observed only in some chromosomes (Machado *et al*., 2021).

**Figure 3:**
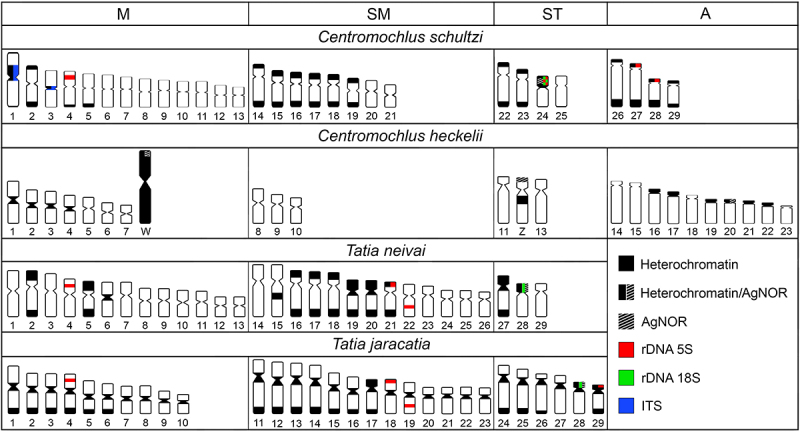
Idiograms representing the karyotypes and locations of heterochromatin, Ag-NORs, 5S rDNA, 18S rDNA, and ITSs in *C. schultzi* compared to *C. heckelii* (Kowalski et al., 2020), *T. neivai* and *T. jaracatia* (Lui *et al*., 2013b).

Simple NORs are a common feature among Auchenipteridae species, with variation in position (terminal or interstitial) and morphology of the chromosomal pair. *Centromochlus heckelii* is the only species of the family with multiple NORs (Kowalski et al., 2020). If we consider the morphology of the chromosomal pair bearing the NORs and the position of the site in comparison with the currently studied *Centromochlus* and *Tatia* species, it is possible to highlight the following aspect: in both *Tatia* species (*T. jaracatia* and *T. neivai*) and in *C. schultzi* the NORs are in subtelocentric pairs, while in *C. heckelii* the NORs are in an acrocentric pair and also in the sex chromosome pair (Table 1, Figure 3). This data demonstrates a greater similarity for this marker between the *Tatia* species and *C. schultzi* than between congener species in *Centromochlus*. In Doradidae, the simple NOR is probably the ancestral feature for most clades, wherein *Platydoras hancockii* Valenciennes 1840 is the only species in the family to present multiple NORs (Takagui et al., 2021).

The ribosomal DNA mapping in Auchenipteridae is limited to a few species (Table 1). Despite the 18S rDNA sites being conserved in relation to the number of carrier pairs, the 5S rDNA is more variable among the studied species of Auchenipteridae. *Centromochlus schultzi* presented the 5S rDNA sites in four chromosomal pairs, in which, the site in pair 3m may be considered a homeologue to the pairs 4m of both *Tatia* species (as reported in Lui *et al*., 2013b) based on the similarities in morphology and location of the sites, as well as the phylogenetic proximity within the Auchenipteridae family. Although there is similarity in the rDNA distribution in the *C. schultzi* karyotype in comparison to the *Tatia* species, *C. schultzi* exhibits 18S/5S rDNAs synteny detected in pair 24st. Therefore, since the 5S rDNA is the most variable chromosomal marker within this fish group (Table 1), it consequently holds significant potential to elucidate the mechanisms involved in the chromosomal evolution of Centromochlinae.

In fish, the standard arrangement of ribosomal sites is usually in distinct chromosomes (Martins and Galetti Jr, 2001; Gornung, 2013). Studies suggest that since these genes are transcribed by different polymerases and the processes occur in distinct nuclear territories (Amarasinghe and Carlson, 1998), the location of ribosomal genes in different chromosomes and positions would be a way to limit the occurrence of adverse rearrangements (Dover, 1986; Martins and Galetti Jr, 1999, 2000; Martins and Wasko, 2004; Diniz et al., 2009). However, several groups of Neotropical fish carry these ribosomal genes in synteny, distant or colocalized. Several recent studies in Siluriformes showed the synteny of these genes (*e.g.*, Baumgärtner et al., 2018; Fonseca et al., 2018; Lorscheider et al., 2018; Takagui et al., 2019; Terra et al., 2019), being considered as a plesiomorphic feature in Tricomycteridae and Loricariidae (Ziemniczak et al., 2012), and an ancestral condition in the sister group of Auchenipteridae, the Doradidae family (Baumgärtner *et al*., 2018; Takagui *et al*., 2019).

Considering this recent proposal made for Doradidae (Takagui et al., 2019), two hypotheses can be made regarding the evolution of this character in the Doradoidea superfamily: (1) the 18S/5S rDNA synteny, detected for the first time in Auchenipteridae in *C. schultzi*, comprises a plesiomorphic state congruent to the proposal of synteny is ancestral in Doradidae; or (2) this synteny in *C. schultzi* should only be interpreted as an apomorphy of the species or a synapomorphy of some Centromochlinae species. We believe that the second hypothesis is more parsimonious and that the study of additional taxa is required to clarify this issue properly.

Considering the proposal by Sarmento-Soares and Martins-Pinheiro (2020) for Centromochlinae, both *Centromochlus* species that have been studied cytogenetically exhibit signs of Robertsonian rearrangements, as indicated by the presence of ITS in *C. schultzi* and the lowest diploid number in *C. heckelii*; whilst *Tatia* species do not present any signs that Robertsonian rearrangements may have played a role during the group’s diversification. However, it is important to note that the possibility of this characteristic being exclusive to *C. heckelii* cannot be ruled out. It is noteworthy that the only *Glanidium* species studied so far had the telomeric sequence mapping performed and no ITS was detected (Lui *et al*., 2015). Another aspect that differs *Centromochlus* and *Tatia* considering the current data is the absence of acrocentric chromosomes in the clade formed by *T. jaracatia* and *T. neivai*, which are observed in both *Centromochlus* species, with *C. heckelii* presenting a larger number of acrocentric chromosomes despite having a smaller diploid number. The 5S/18S rDNA synteny in *C. schultzi* may be another interesting character in this scenario, since this arrangement has not been visualized in the *Tatia* species. It is also worth mentioning that the data related to these genes have not yet been generated for *C. heckelii* (Figure 3). However, the distribution pattern of NORs in *C. schultzi* is more similar to *Tatia* species, since *C. heckelii* presents NORs in an acrocentric pair and on the Z and W chromosomes (multiple sites), while both *Tatia* species (*T. jaracatia* and *T. neivai*) and *C. schultzi* present NORs in only one subtelocentric pair. Although the Z is also a subtelocentric chromosome, it can be clearly distinguished from the NOR-bearing chromosomes of *Tatia* species and *C. schultzi* based on the C-positive heterochromatin blocks (Table 1, Figure 3). These characters need further investigation and will only be better understood with more Centromochlinae taxa being studied.

The cytogenetic data presented here, compared to the limited available data for Centromochlinae, demonstrate an intriguing level of chromosomal variability among *Centromochlus* and *Tatia* species (Figure 3), even when compared to the data available for other genera and species within the family Auchenipteridae. Furthermore, by analyzing a single taxon, unprecedented chromosomal information was generated for Centromochlinae, which when compared to previously published data, makes cytogenetic analyzes even more valuable and promising for uncovering the evolutionary complexities within Centromochlinae. Therefore, it represents a potential tool to support the taxonomy and the allocation of species among the genera of Centromochlinae.

## Supplementary material

The following online material is available for this article:

Figure S1 **-**
Fluorescent *in situ* hybridization with 5S rDNA probes (green) and telomeric probes (red). Chromosomal pairs with ITSs are identified by the number in the karyotype.
