# Supplementary material for: Karyotypic characterization of Centromochlus schultzi Rössel 1962 (Auchenipteridae, Centromochlinae) from the Xingu River basin: New inferences on chromosomal evolution in Centromochlus
Source: Genet Mol Biol. 2024 Mar 25;47(1):e20230105. doi: 10.1590/1678-4685-GMB-2023-0105 (PMC10993310; doi:10.1590/1678-4685-GMB-2023-0105)
Supplement: Figure S1 - [file 1415-4757-GMB-47-1-e20230105-s1.pdf]

Genetics and Molecular Biology
https://doi.org/10.1590/1678-4685-GMB-2023-0105

Supplementary Material to "Karyotypic characterization of *Centromochlus schultzi* Rössel 1962 (Auchenipteridae, Centromochlinae) from the Xingu River basin: new inferences on chromosomal evolution in *Centromochlus*"

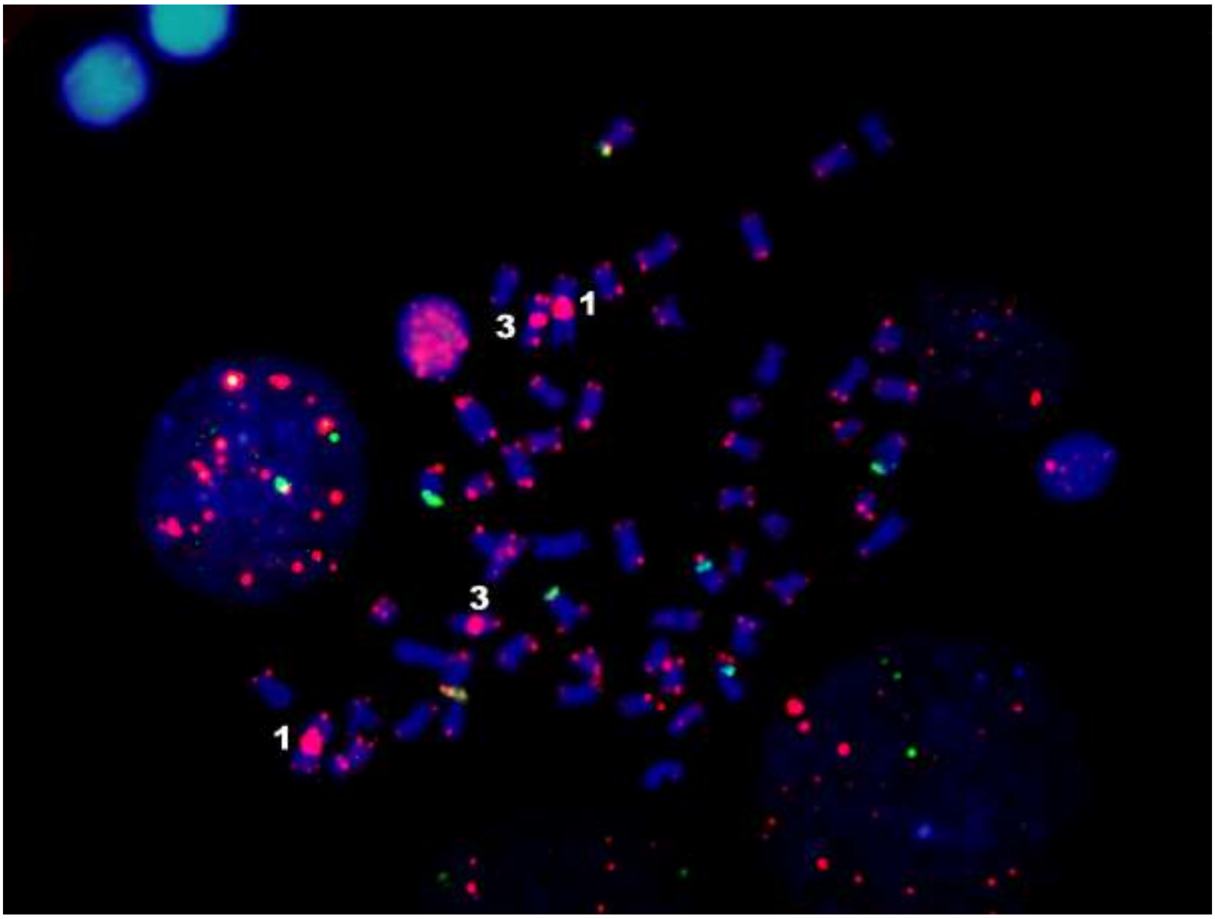

Figure S1 – Fluorescent *in situ* hybridization with 5S rDNA probes (green) and telomeric probes (red). Chromosomal pairs with ITSs are identified by the number in the karyotype.
